# Export of Glandered Horses

**Published:** 1896-05

**Authors:** 


THE *Veterinary Record* refers to the agitation among the English people on the danger of receiving glandered horses through the numerous importations now being made. They quote the Chairman of the Committee on Diseases of the U. S. V. M. A. as to its prevalence in certain localities in our country. The prompt action of Secretary of Agriculture Morton planning for the proper and thorough inspection of these horses before shipment, and directing that shipment may be made only from certain designated ports, will afford Great Britain the necessary safeguards, and preserve this trade for our breeders and dealers.

